# 

**Published:** 1854-10

**Authors:** 


### CONTENTS OF VOLUME VIII.

American Society of Dental Surgeons........ 9
A Dissertation on the Diseases of the Dental Pulp and their Treatment. By C. A. Harris, M. D........ 11
Address of W. H. Goddard—Miss. Association........ 155
Alloys. By G. Watt, M. D., D. D. S.,........ 170
A New Method of Operating on loose or sensative Teeth. By W. A. Helme, D.D.S. 220
Address of Dr. Townsend before the American Society of Dental Surgeons........ 243
Biographical Notice of Dr. Flagg........ 42
Crystal Gold. The manner of using it. By J. Taft, D. D. S........ 89
Case of Dr. Beale and Psycological Effects of Ether. By E. Hartshorne, M. D.... 119
Correspondence........ 145
Case of Remittent Cranial Neuralgia, resulting from Decayed Teeth. By J. Richardson, M. D., D. D. S........ 253
Dental Education. By J. S. Rock, M. D., D. D. S........ 34
Dental Patents........ 49
Dental Abstracts........ 54
Difficult Cases of Extractions. By James Taylor, M. D........ 94
Editorial........ 73
Editorial........ 148
Eleventh Annual Meeting of the Miss. Dental Association........ 183
Editorial........ 228
Editorial........ 355
Gutta Percha Bases for Artificial Teeth........ 225
Gleanings from my Journal of Five Year's Practice--on Exposed Nerves........ 260
Great Dental Patent Case........ 267
Hegienics of Temperance. By S. A. Cartwright, M. D........ 27
Harris on Air Chambers........ 216
Impressions in Plaster of Paris........ 116
Method of Directing Second Dentition. By James Taylor, M. D., D. D. S........ 1
" " " " " " "........ 77
Minutes of the Third Annual Meeting of the Ohio Dental College Association...... 192
Method of avoiding Warping of Plates........ 218
Munro's Address........ 199
Mississippi Valley Association of Dental Surgeons........ 235
Our Position on the Gold Question........ 258
Practical Thoughts on Tooth Drawing. By C, T. Cushman,........ 101
Preparation of Cavities for Filling Teeth. By A. S. Talbert........ 175
Review of Editorial Remarks on Sponge Gold. By J. D. White, by J. T........ 84
Risodontropy. By S. D. Muse........ 91
Rattlesnakes and Delirium Tremens........ 39
Separation of the Teeth. By J. Taft, D. D. S........ 261
The Fifteenth Annual Meeting of the American Society of Dental Surgeons, convened according to Appointment, in the Hall of the Ohio Dental College, in the city of Cincinnati, Ohio, on Thursday, May 8th, 1855........ 238
Treatment of Alveolar Abscess. By Charles W. Ballard, M. D. D. D. S........ 108
The Dental Profession in Europe........ 222
Trial of Allen *vs* Hunter........ 267
Valedictory Address to the Graduating Class of the Ohio College of Dental Surgery, Session of 1854-55. By James Taylor, M. D., D. D. S........ 199

### RECEIPTS FOR THE REGISTER.

| | |
|---|---|
| Dr. M. B. Ross, Newport, Ky., vol. 6 and 7 | $4,00 |
| Dr. Andrews, Yazoo City, Miss., vol. 4 | 2,00 |
| Dr. S. D. Muse, Sharon, Miss., vol. 6 and 7 | 4,00 |
| Dr. P. G. Hamill, Georgetown, Ky., vol. 7 | 2,00 |
| Dr. J. F. Wilson, Greencastle, Ia. vol. 7 | 2,00 |
| Dr. John R. Stark, Independence, Mo., vol. 6, 7 and 8 | 5,00 |
| Dr. D. E. Ellis, Hanover, N. Y., vol. 6 and 7 | 5,00 |
| Dr. F. M. Perry, Barton, Vt., vol. 7 | 2,00 |
| Dr. J. L. Walker, Hopkinsville, Ky., vol. 7 | 2,00 |
| Dr. S. Fleager, Vincennes, Ia., vol. 7 and 8 | 4,00 |
| Drs. S. Hawkins, & T. P. Ely, Union, Ohio, vol. 8 | 2,00 |
| Dr. E. G. Hambleton, Frankfort, Ky., vol. 6 | 2,00 |
| Dr. A. G. Vanderbelt, Crawfordsville, Ia., vol. 8 | 2,00 |
| Dr. J. A. Trousdale, Baton Rouge, La., vol. 8 | 2,00 |
| Dr. C. W. Kelsor, Mount Vernon, O., vol. 8 | 2,00 |
| Dr. Wheeler, Murfreysborough, Tenn., vols. 1, 2, 3, 4, 5, 6 and 7 | 14,00 |
| Dr. M. Reynolds, Louisania, Mo., vol. 8 | 2,00 |
| Dr. Grimes, Dubuque, Iowa, vol. 7 | 2,00 |
| Dr. E. Maynard, Washington City, vols. 4, 5, 6 and 7 | 8,00 |
| Dr. George Motter, Chilicothe, O., vol. 8 | 2,00 |
| Dr. John Fry, Ligonier, Pa., vol. 8 | 2,00 |
| Dr. Ludlow, Indianapolis, Ia., vol 6 and 7 | 4,00 |

### CONTENTS OF THIS (OCTOBER) NUMBER.

ORIGINAL COMMUNICATIONS.

Method of Directing Second Dentition. By James Taylor M. D., D. D. S.............. 1
American Society of Dental Surgeons........................................................ 9

SELECTED ARTICLES.

A Dissertation on the Diseases of the Dental Pulp and their Treatment. By Chapin A. Harris, M. D., D. D. S.......................................................... 11
Hygienics of Temperance, or Water and Alcohol Contrasted on the People Proper. By Samuel A. Cartwright, M. D.................................................... 27
Dental Education By J. S. Rock, M. D., D. D. S........................................ 34
Rattlesnakes and Delirium Tremens............................................................ 39
Biographical Notice of the late Josiah Foster Flagg, M. D., D. D. S................. 42
Dental Patents.................................................................................... 49
Dental Abstracts................................................................................. 54
Editorial........................................................................................... 73

### Baltimore College of Dental Surgery.

### Fourteenth Session, 1854-'55.

CHAPIN A. HARRIS, A. M., M. D.,
Professor of Principles and Practice of Dental Surgery.
THOMAS E. BOND, Jr., A. M., M. D.,
Professor of Special Pathology and Therapeutics.
WASHINGTON R. HANDY, M. D.,
Professor of Anatomy and Physiology.
ALFRED A. BLANDY, M. D.,
Professor of Operative Dentistry.
PHILIP H. AUSTEN, A. M., M. D.,
Professor of Mechanical Dentistry.
REGINALD N. WRIGHT, A. M., M. D.,
Lecturer on Chemistry and Metallurgy.

The regular course of lectures will commence on the 1st of November, and close the 1st of March. The Infirmary, Mechanical and Dissecting rooms will be opened on the first Monday in October, during which month there will be occasional lectures from each of the professors.

It is important that every student should, if possible, be present during this preliminary month. The Infirmary will continue open during the summer for the benefit of those students who remain in the city between their first and second courses of lectures.

Candidates presenting themselves for examination must have attended two full courses of lectures. Certificates of four years dental practice, or of one full course in any respectable Dental or Medical College will be received as equivalent to the *first* Course.

Tickets for the Course, $100. Dissecting Ticket, (optional,) $10. Diploma Fee, $30. Marticulation Fee, $5.

Rooms and board may be obtained at from $2 50 to $4 00 per week.

Text Books. Harris' Principles and practice of Dental Surgery, 5th Edition ; Bond's Dental Medicine, 2d Edition ; Handy's Anatomy ; Carpenter's Physiology ; Mutter's Liston's Surgery ; Jourdain's Surgical Diseases of the Mouth ; Mitchell's or Dunglison's Therapeutics and Materia Medica ; Fowne's, Graham's or Turner's Chemistry ; Wood's or Watson's Practice of Medicine.

P. H. AUSTEN, Dean, 84 *Sharp Street, Baltimore.*

### THE OHIO COLLEGE OF DENTAL SURGERY.

**Session of 1854–'55.**

The new College Edifice is ready for use and well adapted in every way.

The regular course of lectures in this institution, commences on the first Monday of November, and closes on Wednesday, succeeding the third Monday of February.

**FACULTY.**

JAMES TAYLOR, M. D., D. D. S.
*Professor of the Principles and Practice of Dental Surgery.*

THOMAS WOOD, M. D.,
*Professor of Anatomy and Physiology.*

J. B. SMITH, M. D.,
*Professor of Pathology and Therapeutics.*

JONATHAN TAFT, D. D. S.,
*Professor of Operative Dentistry.*

H. R. SMITH, D. D. S.,
*Professor of Mechanical Dentistry.*

GEORGE M. KELLOGG, M. D.,
*Lecturer on Chemistry.*

**EXAMINING COMMITTEE.**

PHYSICIANS.

PROF. L. M. LAWSON, M. D., *Cincinnati, O.*
PROF. JOHN DAVIS, M. D., *Cincinnati, O.*

DENTISTS.

J. P. ULLREY, D. D. S., *Rising Sun, Ia.*

J. G. HAMILL, D. D. S., *Georgetown, Ky.*

C. H. QUINLAN, D. D. S., *Chicago, Ill.*

JANITOR.

MR. JOSEPH EDWARDS.

**TERMS OF ADMISSION.**

Tickets are considered as cash, and when not paid for in advance, a note to the Faculty, with approved security, will be required.

| | |
|---|---|
| Tickets for the entire course, and admissions to dissections, | $100 00 |
| Marticulation Ticket, | 5 00 |
| Diploma Fee, | 25 00 |

Good and respectable Board can be had at from $2,50 to $3,50 per week.

JAMES TAYLOR, Dean.

---

### Removal.

W. Z. REES Respectfully informs his friends and patrons, that he has REMOVED his Manufactory to Sixth Street, between Walnut and Vine, South side. A general assortment of DENTAL and SURGICAL INSTRUMENTS always on hand and made to order. Also a general assortment of PEARL HANDLES and MIRRORS always on hand

